# Cognitive-Behavioral Therapy Versus Traditional Approaches in Treating Anxiety-Depression Among International Medical Students

**DOI:** 10.1192/j.eurpsy.2025.1301

**Published:** 2025-08-26

**Authors:** M. S. Artemieva, A. D. Shadrikova, A. G. Lazukova, V. P. Sokolov, N. O. Danilina

**Affiliations:** 1 Psychiatry and Medical Psychology, RUDN University, Moscow, Russian Federation

## Abstract

**Introduction:**

Adapting to a new socio-cultural environment presents a major challenge for African medical students, often leading to the development of anxiety-depressive disorders. To date, a number of therapeutic approaches including psychotherapy, educational programmes and pharmacological treatments are available, but their comparative effectiveness for this group remains poorly researched.

**Objectives:**

The present study aims to comparatively evaluate different methods of psycho-emotional state correction.

**Methods:**

The study included 72 African undergraduate medical students (age range 18-23 years, 54.9% female, 45.1% male).

Participants were assigned to three therapeutic groups. Statistical processing with post-hoc test and Bonferroni correction confirmed the benefit of the psychotherapeutic approach in reducing levels of anxiety and depression (p≤0.01). An 8-week cognitive-behavioural therapy programme demonstrated the greatest effectiveness. A one-day educational workshop showed moderate efficacy. Medication therapy with antidepressants for 8 weeks resulted in minimal improvement.

**Results:**

Participants were assigned to three therapeutic groups. Statistical processing with post-hoc test and Bonferroni correction confirmed the benefit of the psychotherapeutic approach in reducing levels of anxiety and depression (p≤0.01). An 8-week cognitive-behavioural therapy programme demonstrated the greatest effectiveness. A one-day educational workshop showed moderate efficacy. Medication therapy with antidepressants for 8 weeks resulted in minimal improvement.

**Conclusions:**

Cognitive-behavioural therapy demonstrated the greatest efficacy in the correction of anxiety-depressive disorders in international medical students, outperforming both educational and pharmacological interventions. Subjective satisfaction of participants was also higher in the psychotherapy group, indicating that it is appropriate to prioritise the use of non-pharmacological methods in this population.

**Disclosure of Interest:**

None Declared

